# Light-Dependent Regulation of Drought Tolerance in Cucumber Plants by Melatonin

**DOI:** 10.3390/ijms27156898

**Published:** 2026-08-01

**Authors:** Ekaterina V. Boyko, Irina F. Golovatskaya, Maksat Kadyrbaev, Liliya V. Kolomeichuk, Olga K. Murgan, Darya P. Kozhemyakina, Evgeniy G. Boyko

**Affiliations:** 1 Department of Biotechnology and Bioinformatics, Higher Engineering School of Agricultural Biotechnology, National Research Tomsk State University, 634050 Tomsk, Russia; golovatskaya.irina@mail.ru (I.F.G.); kadyrbaev.maks@mail.ru (M.K.); kolomeychuklv@mail.ru (L.V.K.); reborn_rinni@mail.ru (O.K.M.); cgd2695@gmail.com (D.P.K.); boykoegagrobiotek@gmail.com (E.G.B.); 2 Laboratory of Cell and Microfluidic Technologies, Faculty of Pharmacy, Siberian State Medical University, 634050 Tomsk, Russia

**Keywords:** *Cucumis sativus*, melatonin, drought, light, photosynthesis, antioxidant system, gene expression

## Abstract

Water availability, light spectral composition, and phytohormones jointly regulate plant morphogenesis and photosynthesis. Light quality can modify stress responses by altering endogenous regulators such as melatonin. However, it remains unclear whether light spectrum modulates melatonin-mediated protection under osmotic stress. Here, we investigated the effects of 1 μM melatonin on cucumber (*Cucumis sativus* L.) subjected to polyethylene glycol 6000 (PEG-6000)-induced osmotic stress under three distinct photosynthetically active radiation (PAR) spectral compositions. Our results indicate that PAR spectral composition determines both the magnitude and the primary physiological targets of the melatonin response. Under red-enriched illumination, melatonin predominantly enhanced photosystem II performance, evidenced by increased effective quantum yield of PSII [Y(II)] and electron transport rate (ETR), reduced lipid peroxidation, and altered expression of genes involved in antioxidant defense, melatonin biosynthesis, and auxin signaling. Specifically, expressions of *SOD*, *APX*, and *ARF* were upregulated, while *SNAT* expression was downregulated. Under blue-enriched illumination, melatonin effects were mainly associated with stomatal regulation: stomata remained more open, stomatal area and density increased, proline accumulated, and guaiacol peroxidase activity rose, collectively supporting water balance and gas exchange. These findings suggest that melatonin functions as a light-dependent regulator of cucumber responses to PEG-induced osmotic stress, with its physiological and molecular effects being modulated by the spectral quality of PAR.

## 1. Introduction

During ontogenesis, plants are continuously exposed to a wide range of abiotic and biotic stressors, both in natural ecosystems and in agroecosystems. Drought is one of the most severe environmental factors affecting plant productivity [1]. Hydrological, agricultural, and meteorological droughts restrict water availability for major food crops in different ways, directly affecting plant growth and yield [1,2,3]. Depending on the plant species, developmental stage, stress intensity, and duration, water deficit disrupts cellular metabolism, thereby inhibiting plant growth and development. Drought-induced yield losses may range from 30% to 90%, depending on the crop species and environmental conditions [3]. To survive under water-deficit conditions, plants employ adaptation and acclimation mechanisms involving changes in osmotic regulation, root and leaf growth, stomatal behavior, photosynthetic activity, and antioxidant defense [1,2,3]. Nevertheless, despite the diversity of these responses, most major agricultural crops remain highly sensitive to drought.

Water deficit in agricultural crops may result from a complex combination of factors, including insufficient precipitation, high temperature, excessive irradiance, dry winds, inadequate irrigation, high electrical conductivity caused by solutes in the soil solution, and improper fertilizer application [4]. In addition, the concept of physiological drought, or pseudodrought, describes situations in which soil moisture is sufficient, but water uptake by roots is impaired by unfavorable soil conditions, including low soil temperature, flooding, or the presence of substances that restrict root water absorption [4,5].

In recent years, increasing attention has been paid to the role of light quality in plant stress tolerance. With respect to biotic stressors, red-light illumination has been shown to enhance cucumber resistance to powdery mildew (*Sphaerotheca fuliginea*) by promoting salicylic acid biosynthesis and activating the expression of salicylic acid- and defense-related genes [6]. Cultivation of tomato plants under blue light increased resistance to gray mold by enhancing proline accumulation, polyphenolic compound content, and antioxidant enzyme activity [7]. Evidence has also accumulated that exposure to light of different spectral compositions can increase plant tolerance to abiotic stressors. For example, pretreatment with combined red and blue light enhanced drought tolerance in lemon balm plants [8]. Short-term exposure to blue light increased potato tolerance to chloride salinity through the accumulation of non-enzymatic antioxidant defense components, including carotenoids and proline [9]. Red and blue light have also been reported to enhance cadmium tolerance in rice seedlings [10].

Another approach to increasing plant productivity and tolerance to adverse environmental conditions involves the application of hormone-like compounds [11,12]. Melatonin is of particular interest in this context. It is a phylogenetically ancient, multifunctional indole molecule widely distributed among organisms from different taxonomic groups. Melatonin and its degradation products act as effective antioxidants that reduce the levels of reactive oxygen species and reactive nitrogen species [13,14]. Under stress conditions, exogenous melatonin promotes plant growth and viability, maintains photosynthetic performance, and stimulates the accumulation of sucrose and proline [15,16,17].

Previous studies have shown that melatonin content depends on light spectral composition and that its physiological effects may vary under different light conditions [18,19,20]. However, it remains insufficiently understood whether light spectral composition changes the predominant physiological and molecular targets of exogenous melatonin during water-deficit stress. Cucumber (*C. sativus* L.) is considered a drought-sensitive crop species and is therefore widely used as a model for investigating physiological and molecular responses to water deficit [21,22].

We therefore hypothesized that signaling processes associated with different spectral compositions of light may interact with melatonin-mediated regulation of plant physiological responses under normal and stress conditions. The present study aimed to determine how the spectral composition of photosynthetically active radiation modifies melatonin-mediated responses of cucumber plants exposed to osmotic stress induced by polyethylene glycol 6000 (PEG-6000).

## 2. Results

### 2.1. Dependence of Melatonin-Mediated Growth Responses of Cucumber Seedlings and Plants on Light Quality and Drought

**Figure 1:**
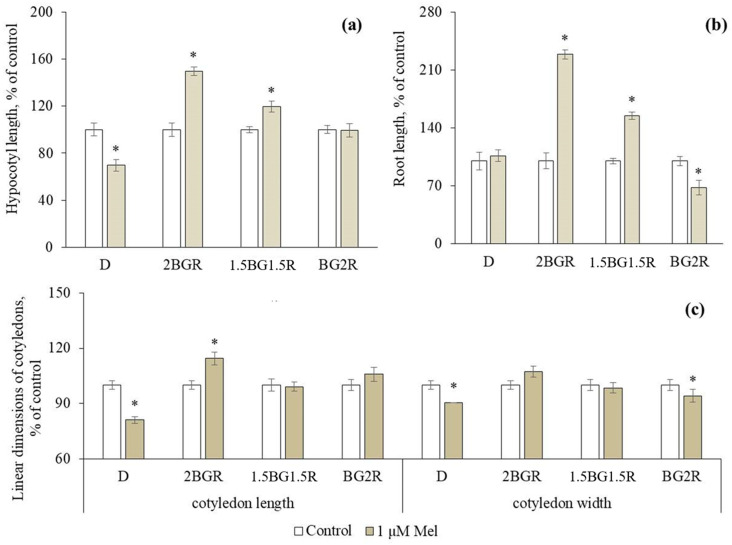
Effect of melatonin on the morphogenesis of *C. sativus* seedlings in darkness and under light of different spectral compositions: (**a**) hypocotyl length; (**b**) root length; (**c**) cotyledon length and width. Note: D, darkness; the photon flux density ratios of blue:green:red light were 2:3:1 (2BGR), 1.5:3:1.5 (1.5BG1.5R), and 1:3:2 (BG2R). Asterisks (*) indicate statistically significant differences compared with the corresponding control (*p* ≤ 0.05).

The effect of melatonin on cucumber seedling growth depended heavily on the light regime. Melatonin stimulated root and hypocotyl growth under the 1.5BG1.5R and 2BGR spectra, whereas it inhibited root growth under BG2R (Figure 1a,b). In darkness, melatonin suppressed cotyledon blade growth, whereas under 2BGR it stimulated cotyledon development (Figure 1c).

**Figure 2:**
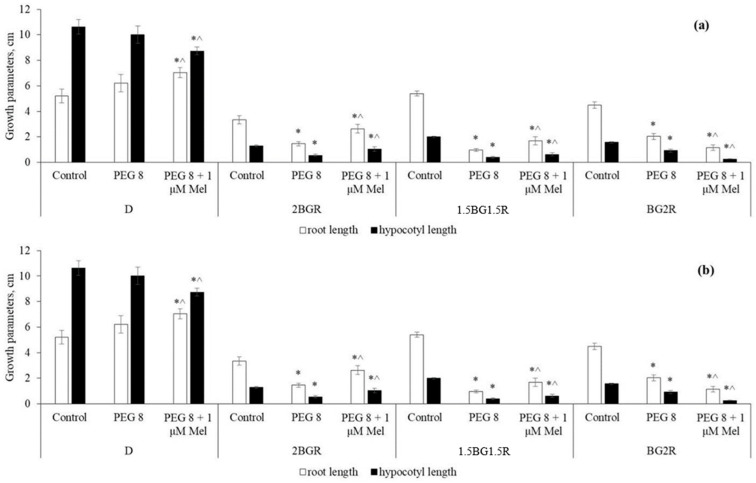
Effect of melatonin on the morphogenesis of *C. sativus* seedlings under drought stress (PEG 8), in darkness, and under light of different spectral compositions: (**a**) root and hypocotyl length; (**b**) cotyledon length and width. Note: Asterisks (*) indicate statistically significant differences compared with the corresponding control within the same light regime (*p* ≤ 0.05); carets (^) indicate statistically significant differences compared with PEG 8 within the same light regime (*p* ≤ 0.05).

Analysis of the obtained data revealed a pronounced negative effect of drought on cucumber seedling morphogenesis (Figure 2). Drought treatment with PEG 8 reduced the length of axial organs by 56% compared with optimal water supply (the control). Seedling growth under drought conditions strongly depended on the light spectral composition. Increasing the proportion of the red spectral region inhibited root and hypocotyl elongation. The most severe negative effect of drought—a 5.0–5.6-fold decrease relative to the control—was observed when plants were cultivated under 1.5BG1.5R. A twofold increase in the proportion of red light (under BG2R) reduced root and hypocotyl length under drought by 54% and 41%, respectively, compared with the control (Figure 2).

Root application of melatonin exacerbated the negative impact of drought in darkness, but alleviated the inhibitory effect of the stressor on root elongation under 2BGR and 1.5BG1.5R, stimulating root growth by 80% and 75%, respectively, compared with drought treatment alone (PEG 8). However, under BG2R light, melatonin intensified the inhibition of root elongation by 43% compared with seedlings exposed to drought alone (Figure 2a). The effects of melatonin observed for roots under various light spectra were mirrored in the hypocotyl: melatonin mitigated the negative impact of drought under 2BGR and 1.5BG1.5R. In contrast, in darkness and under an increased proportion of red light, melatonin further suppressed hypocotyl elongation (Figure 2).

At early stages of cucumber ontogenesis, cotyledons serve as the primary photosynthetic organs. Drought negatively affected cotyledon blade formation, with the ratio of different spectral regions influencing the magnitude of the growth response. The least pronounced negative effect of drought on cotyledon growth was observed in darkness, where linear parameters decreased by 15% compared with the control. Cultivation of seedlings under 2BGR during drought reduced cotyledon length by 39% and width by 43%. Under BG2R and drought conditions, the inhibition of cotyledon blade elongation in length and width reached 37% and 42%, respectively (Figure 2b). Notably, the inhibitory effect of drought on cotyledons was comparable between the 2BGR and BG2R variants, indicating a limited influence of the PAR spectral composition on cotyledon tolerance to osmotic stress at this developmental stage. The strongest negative effect of drought was observed under 1.5BG1.5R; under these conditions, cotyledons were severely underdeveloped and remained enclosed within the seed coat (Figure 2b).

**Figure 3:**
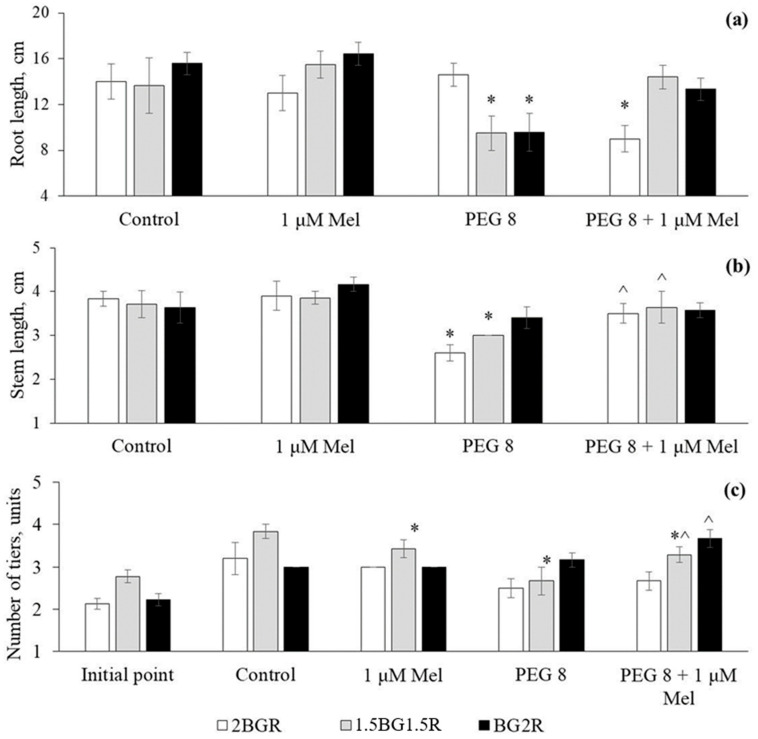
Effect of melatonin on: (**a**) root length; (**b**) stem length; (**c**) the number of leaf tiers in 21-day-old *C. sativus* plants under PEG-induced drought stress and light of different spectral compositions. Note: Asterisks (*) indicate statistically significant differences compared with the corresponding control within the same light regime (*p* ≤ 0.05); carets (^) indicate statistically significant differences compared with PEG 8 within the same light regime (*p* ≤ 0.05).

As the proportion of red light in the light flux increased, the leaf surface area of the first and second leaf tiers decreased in control cucumber plants, while stem and root growth parameters remained stable (Figure 3a,b). Three-day melatonin treatment did not significantly affect the growth of axial organs (Figure 3); however, transfer of plants to drought conditions (PEG 8) significantly slowed root and stem elongation. The strongest inhibition of stem elongation, 32%, was observed in plants grown under 2BGR, whereas under 1.5BG1.5R it reached 19% (Figure 3b). The addition of melatonin to the nutrient medium under drought conditions reduced root length but increased stem length in cucumber plants grown under blue-enriched PAR (2BGR). When the proportion of the red spectral region was increased, melatonin stimulated root growth under 1.5BG1.5R and BG2R and stem growth under 1.5BG1.5R, thereby alleviating the negative effect of drought (Figure 3a,b).

Selective 1.5BG1.5R light promoted the formation of a greater number of tiers in cucumber plants (Figure 3c). The number of tiers at the moment of transfer to drought conditions was used as the initial baseline for shoot development. The addition of 1 µM melatonin to the nutrient medium inhibited the initiation of new tiers by 10% under 1.5BG1.5R. A negative effect of drought was observed in cucumber plants growing under 2BGR and 1.5BG1.5R, where the number of tiers decreased by 21% and 30%, respectively. The addition of melatonin to the nutrient medium under drought conditions (PEG 8 + 1 µM Mel) accelerated the initiation of new tiers under 1.5BG1.5R and BG2R by 21% and 16%, respectively, compared with the stress treatment alone (Figure 3c).

**Figure 4:**
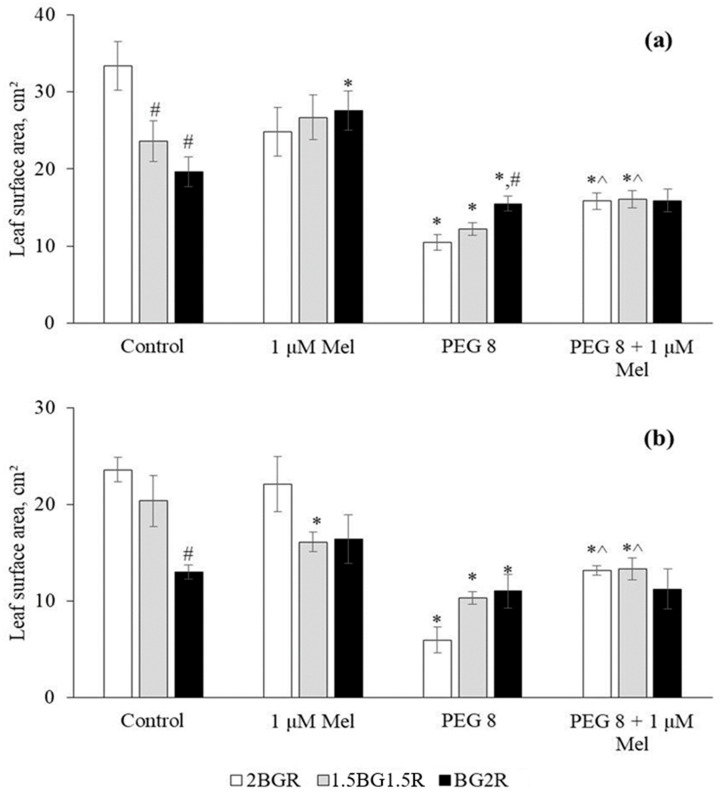
Effect of melatonin on: (**a**) the area of first-tier leaves; (**b**) second-tier leaves in 21-day-old *C. sativus* plants under drought stress (PEG 8) and light of different spectral compositions. Note: Asterisks (*) indicate statistically significant differences compared with the corresponding control within the same light regime (*p* ≤ 0.05); carets (^) indicate statistically significant differences compared with PEG 8 within the same light regime (*p* ≤ 0.05); number signs (#) indicate statistically significant differences among light regimes within the control treatment (*p* ≤ 0.05).

Cultivation of cucumber plants under blue-enriched light (2BGR) combined with a 3-day root application of 1 µM melatonin reduced the surface area of first-tier leaves (Figure 4a). Conversely, an increase in the proportion of red light (1.5BG1.5R) reduced the surface area of second-tier leaves (Figure 4b). Drought significantly inhibited the expansion of both first-tier (Figure 4a) and second-tier leaves (Figure 4b), with the most pronounced decreases observed under 2BGR and 1.5BG1.5R. The addition of 1 µM melatonin partially alleviated the negative impact of drought in plants grown under these PAR spectral regimes (Figure 4).

Analysis of cucumber leaf surface morphology showed that the highest stomatal density per unit epidermal area was characteristic of leaves formed under 1.5BG1.5R, whereas the lowest stomatal density was observed under BG2R (Table 1). Concurrently, leaves formed under 2BGR exhibited the largest stomatal area, while those formed under 1.5BG1.5R had the smallest stomatal area. The patterns observed in our study are consistent with data previously obtained for cucumber plants grown under red, blue, or combined light [23]. Those authors reported the highest stomatal density under mixed light (70% red and 30% blue) and the lowest stomatal density under red light, while also demonstrating light-dependent variations in stomatal dimensions [23].

Root application of melatonin in our experiment increased stomatal density per unit leaf area under BG2R (Table 1), whereas stomatal area remained unchanged relative to the control. Under 1.5BG1.5R, melatonin increased the size of the stomatal aperture and, consequently, the total stomatal area in leaves.

Drought reduced stomatal density per unit leaf surface area by 12%, 25%, and 14% in leaves formed under 2BGR, 1.5BG1.5R, and BG2R, respectively (Table 1). Under blue-enriched light (2BGR), drought decreased stomatal area and caused a twofold reduction in stomatal aperture width. In contrast, under BG2R, drought increased stomatal area by 53% and stomatal aperture area by 20%. Under 1.5BG1.5R, a slight increase in stomatal area was observed, whereas stomatal aperture area remained unchanged. The addition of melatonin to the nutrient medium under drought conditions reduced stomatal density per unit epidermal area under 2BGR. Under BG2R, however, stomatal density increased, whereas under 1.5BG1.5R this parameter remained unaffected. Melatonin maintained stomata in a more open state under 2BGR and 1.5BG1.5R, but slightly reduced their aperture under BG2R (Table 1).

### 2.2. Effect of Melatonin on Biochemical Processes in Cucumber Plants Depending on Light Spectral Composition and Drought

**Figure 5:**
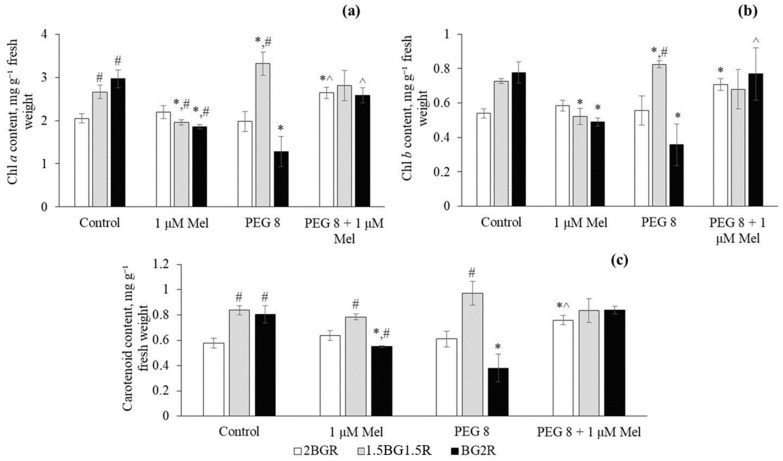
Effect of melatonin on photosynthetic pigment content in first-tier leaves of 21-day-old *C. sativus* plants under drought stress (PEG 8) and light of different spectral compositions. (**a**) chlorophyll *a* content; (**b**) chlorophyll *b* content; (**c**) carotenoid content. Note: Asterisks (*) indicate statistically significant differences compared with the corresponding control within the same light regime (*p* ≤ 0.05); carets (^) indicate statistically significant differences compared with PEG 8 within the same light regime (*p* ≤ 0.05); number signs (#) indicate statistically significant differences among light regimes within the control treatment (*p* ≤ 0.05).

The content of photosynthetic pigments in first-tier leaves increased with an increasing proportion of the red spectral region (Figure 5, control). The addition of 1 µM melatonin reduced the content of all photosynthetic pigment groups per unit leaf mass under 1.5BG1.5R and BG2R, which was likely primarily associated with the increase in the surface area of first-tier leaves. Drought increased the content of all pigment groups under 1.5BG1.5R, but decreased pigment levels in plants grown under BG2R. Under drought conditions, melatonin increased pigment concentrations under blue-enriched light (2BGR) and restored them to the control levels under 1.5BG1.5R and BG2R.

**Figure 6:**
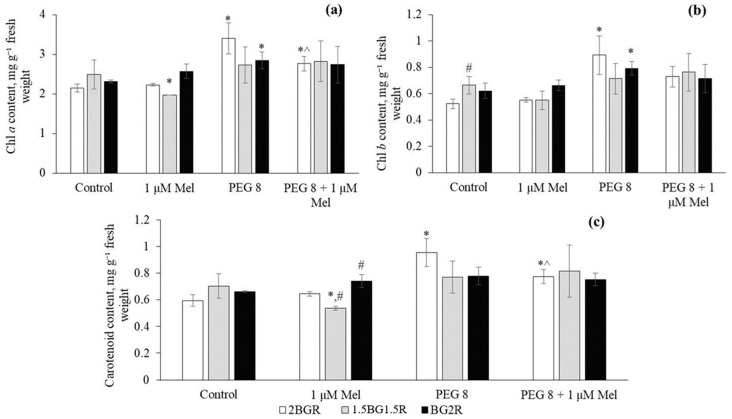
Effect of melatonin on photosynthetic pigment content in second-tier leaves of 21-day-old *C. sativus* plants under drought stress (PEG 8) and light of different spectral compositions. (**a**) chlorophyll *a* content; (**b**) chlorophyll *b* content; (**c**) carotenoid content. Note: Asterisks (*) indicate statistically significant differences compared with the corresponding control within the same light regime (*p* ≤ 0.05); carets (^) indicate statistically significant differences compared with PEG 8 within the same light regime (*p* ≤ 0.05); number signs (#) indicate statistically significant differences among light regimes within the control treatment (*p* ≤ 0.05).

The level of chlorophyll *b* in second-tier leaves was higher in control cucumber plants grown under 1.5BG1.5R than under BG2R (Figure 6b). Conversely, a slight increase in carotenoid content (12%) was observed in plants grown under BG2R (Figure 6c). Treatment with 1 µM melatonin reduced chlorophyll *a* and carotenoid levels under 1.5BG1.5R. Previously, using another plant model, we established the effect of melatonin on the senescence of leaf discs and isolated cotyledons of *Lychnis chalcedonica* L. under selective light [24]. Specifically, melatonin was shown to increase pigment resistance to photooxidation in leaf discs and isolated cotyledons under both blue and red light [24].

Drought, which inhibited growth processes in second-tier leaves, caused an increase in photosynthetic pigment content (Figure 6) in leaves under 2BGR and BG2R relative to the control. However, melatonin reduced pigment levels relative to those in plants grown under drought conditions under 2BGR (Figure 6), which may also be associated with growth and biochemical processes.

Changes in pigment content were accompanied by alterations in the functioning of the photosynthetic apparatus. A general trend toward decreased efficiency parameters—such as Y(II), ETR, and qP—was observed across all light variants after 3 days of drought exposure. The addition of melatonin to the nutrient medium under drought conditions mitigated this adverse effect. Notably, the effect of melatonin was specific to the light regime. In the leaves of plants grown under drought conditions and BG2R, melatonin exerted a moderate stimulatory effect on Y(II), ETR, and NPQ, suggesting its role as a signaling molecule that optimizes photosynthetic performance under a higher proportion of red light (Table 2).

**Figure 7:**
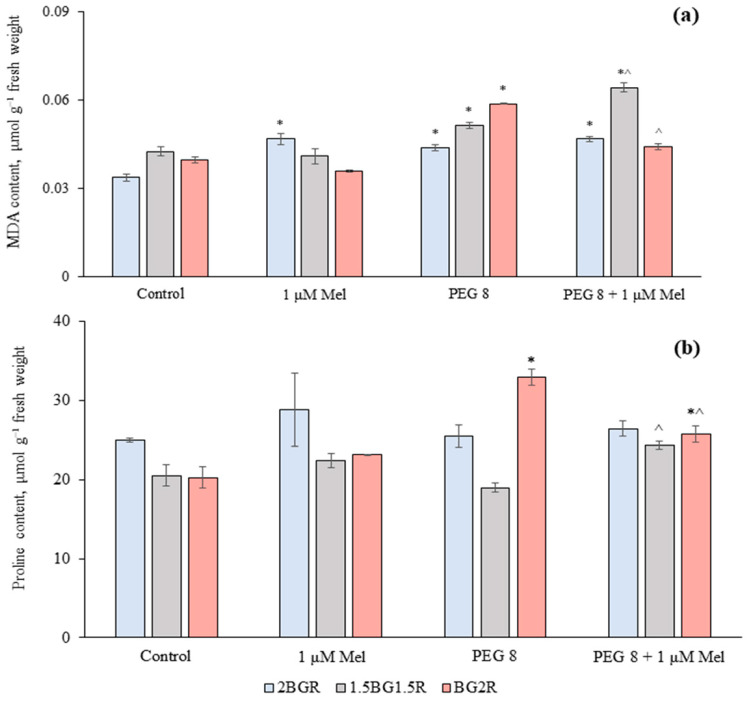
Effect of melatonin on: (**a**) lipid peroxidation intensity; (**b**) proline level in leaves of 21-day-old *C. sativus* plants under drought stress (PEG 8) and light of different spectral compositions. Note: Asterisks (*) indicate statistically significant differences compared with the corresponding control within the same light regime (*p* ≤ 0.05); carets (^) indicate statistically significant differences compared with PEG 8 within the same light regime (*p* ≤ 0.05).

Changes in photosynthetic reactions occurred against the background of altered lipid peroxidation (LPO) intensity. The malondialdehyde (MDA) content in control cucumber plants grown under 2BGR was lower than in those grown under 1.5BG1.5R and BG2R. The addition of melatonin to the nutrient medium increased LPO levels under 2BGR but decreased them under BG2R. Drought increased LPO intensity by 20%, 30%, and 47% under 1.5BG1.5R, 2BGR, and BG2R, respectively, compared with the control (Figure 7). The addition of 1 µM melatonin under drought conditions alleviated this negative effect under 2BGR and BG2R. The combined effect of BG2R and melatonin under drought conditions resulted in the most pronounced reduction in oxidative stress (Figure 7a).

Free proline content exhibited a dependence on light spectral composition. An increase in proline levels was observed under 2BGR. In response to 3 days of drought exposure, proline levels increased under BG2R, whereas the addition of melatonin under stress conditions restored this parameter to the control level (Figure 7b).

**Figure 8:**
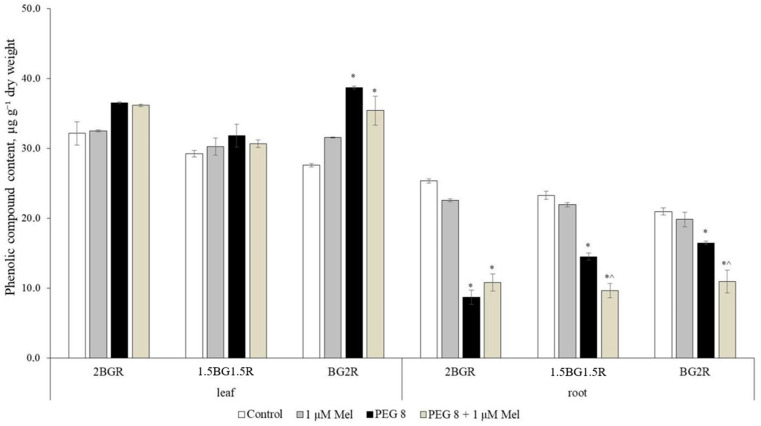
Effect of melatonin on the total content of phenolic compounds in leaves and roots of 21-day-old *C. sativus* plants under drought stress (PEG 8) and light of different spectral compositions. Note: Asterisks (*) indicate statistically significant differences compared with the corresponding control within the same light regime (*p* ≤ 0.05); carets (^) indicate statistically significant differences compared with PEG 8 within the same light regime (*p* ≤ 0.05).

The total content of phenolic compounds also depended on lighting conditions. The lowest content of phenolic compounds in cucumber leaves and roots was observed under BG2R. Drought increased the level of phenolic compounds in cucumber leaves under 2BGR and BG2R but reduced it in roots under all light regimes by 38%, 66%, and 21% for 1.5BG1.5R, 2BGR, and BG2R, respectively. The addition of melatonin under drought conditions (PEG 8 + 1 µM Mel) led to a further decrease in phenolic compound content in roots under 1.5BG1.5R and BG2R compared with PEG 8 alone (Figure 8).

**Figure 9:**
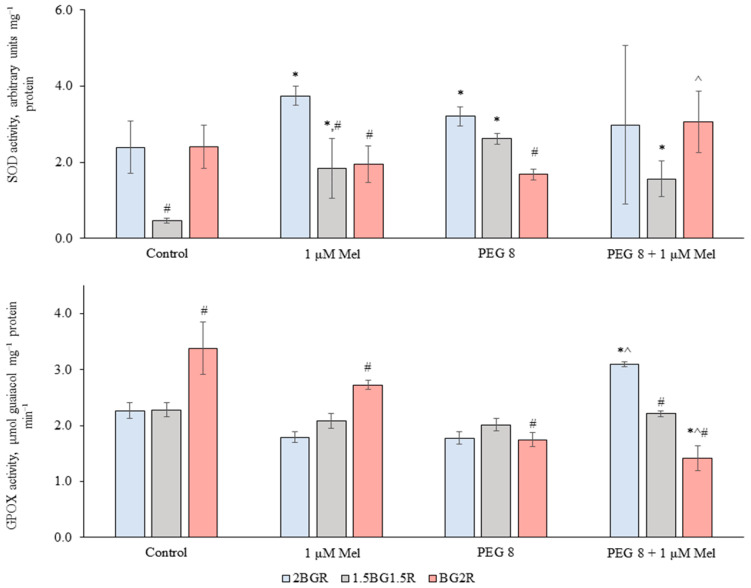
Effect of melatonin on SOD and GPOX activity in leaves of 21-day-old *C. sativus* plants under PEG-induced drought stress and light of different spectral compositions. Note: Asterisks (*) indicate statistically significant differences compared with the corresponding control within the same light regime (*p* ≤ 0.05); carets (^) indicate statistically significant differences compared with PEG 8 within the same light regime (*p* ≤ 0.05); number signs (#) indicate statistically significant differences among light regimes within the control treatment (*p* ≤ 0.05).

Superoxide dismutase (SOD) activity was significantly higher in control plants grown under light with a higher proportion of blue or red light compared to those grown under 1.5BG1.5R. The addition of melatonin to the nutrient medium increased SOD activity under 1.5BG1.5R and 2BGR. Drought activated the enzyme under 1.5BG1.5R and 2BGR but inhibited it under BG2R. Melatonin treatment under stress conditions reduced SOD activity under 1.5BG1.5R and increased it under BG2R (Figure 9).

Peroxidase (GPOX) activity also depended on cultivation conditions; this parameter increased under light with a higher proportion of the red spectral region. Melatonin reduced peroxidase activity under all studied light regimes. Stress exposure decreased peroxidase activity under 2BGR and BG2R, whereas the addition of melatonin under PEG 8 increased enzyme activity by 74% under 2BGR (Figure 9).

### 2.3. Protective Effect of Root-Applied Melatonin on the Regulation of Gene Expression in Cucumber Plants Under Drought Stress

**Figure 10:**
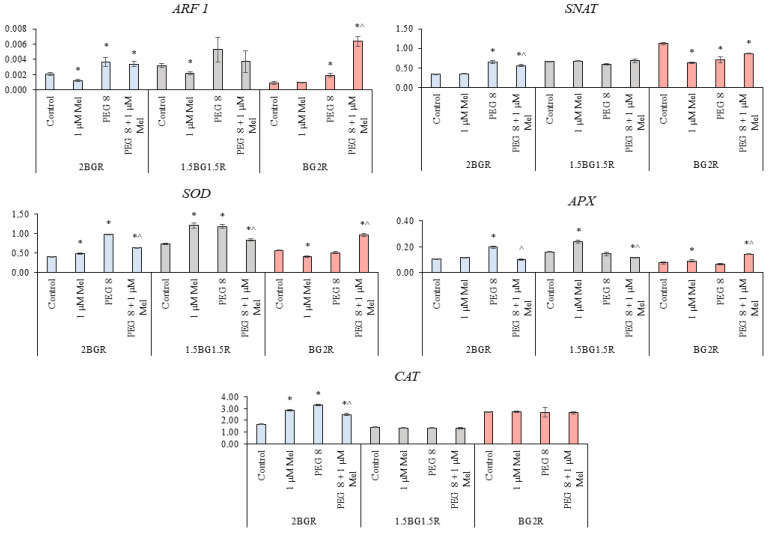
Effect of root-applied melatonin on the expression of genes encoding antioxidant defense enzymes and genes associated with melatonin biosynthesis and auxin response in leaves of 21-day-old cucumber plants under PEG-induced drought stress and light of different spectral compositions. Note: *indicates statistically significant differences compared with the corresponding control within the same light regime (*p* ≤ 0.05); ^ indicates statistically significant differences compared with PEG 8 within the same light regime (*p* ≤ 0.05).

Light-dependent expression was observed for *ARF1*, *SNAT*, *SOD*, *APX*, and *CAT*. The highest expression levels under light with a higher proportion of red light, BG2R, were recorded for *SNAT* and *CAT*, whereas the lowest levels were observed for *ARF1* and *APX* compared with 1.5BG1.5R. Under blue-enriched light, expression levels of all studied genes were low compared with 1.5BG1.5R (Figure 10).

Melatonin treatment altered gene expression in a spectrum-dependent manner. Under 1.5BG1.5R and 2BGR, melatonin reduced *ARF1* transcript abundance. Under the red-enriched BG2R regime, melatonin decreased *SNAT* expression. Conversely, melatonin upregulated antioxidant-defense genes: *SOD* and *APX* transcripts increased under 1.5BG1.5R, and *CAT* expression increased under 2BGR; however, *SOD* expression declined under BG2R following melatonin application (Figure 10).

Drought alone modulated gene expression, and this modulation depended on light quality. *ARF1* was induced by drought across all light regimes. *SNAT* expression decreased under 1.5BG1.5R but increased under 2BGR. Antioxidant-gene responses were spectrum-specific: *SOD* expression rose under 1.5BG1.5R and 2BGR, whereas *APX* and *CAT* were induced by drought only under BG2R (Figure 10).

The combined treatment (drought + melatonin) revealed pronounced spectral dependence. Under red-enriched BG2R light, melatonin applied during drought elicited the coordinated upregulation of key antioxidant genes, *SOD* and *APX*. In contrast, under blue-enriched 2BGR light, the combined treatment suppressed *SOD*, *APX*, and *CAT* expression. Under the intermediate spectrum (1.5BG1.5R), there was a general tendency toward reduced *SOD* and *APX* expression with the combined treatment, while *ARF1* showed a marked induction under BG2R (Figure 10).

## 3. Discussion

Throughout ontogenesis, plants are frequently exposed to adverse environmental factors. Plants at early developmental stages are particularly vulnerable to stress, justifying our focus on cucumber seedlings. Because growth processes rely on cell and organ expansion driven by water uptake, water availability is considered one of the most limiting environmental factors. Therefore, we examined the effects of exogenous melatonin and light spectral composition on the morphogenesis of cucumber seedlings under drought stress.

Drought is one of the most severe environmental factors limiting plant growth and productivity while directly affecting crop yield [1,3]. Recent studies have shown that light spectral composition can significantly influence plant stress tolerance; for instance, blue and red light prevented H_2_O_2_ accumulation under NaCl treatment in wheat plants [25], while blue light enhanced the tolerance of wheat seedlings to excess copper and cadmium ions [26]. In recent decades, increasing attention has been paid to melatonin as a multifunctional signaling molecule capable of modulating plant stress responses [13,14]. However, it remains unclear how light spectral composition, as a fundamental environmental factor, affects the efficiency and direction of melatonin-mediated protection. Our study demonstrates for the first time that light quality determines the primary physiological targets of exogenous melatonin under water deficit, suggesting a light-dependent regulation of drought tolerance in cucumber (*C. sativus* L.) plants. Of particular importance is the identification of two qualitatively different strategies of melatonin-mediated protection, which are deployed depending on the ratio of red to blue spectral regions.

In this study, cucumber seedling development was shown to depend heavily on both light quality and water availability. The least pronounced negative effect of drought was observed in seedlings grown in darkness, which may be attributed to darkness-induced stomatal closure and, consequently, reduced transpirational water loss. Conversely, the predominance of either the blue-enriched (2BGR) or red-enriched (BG2R) region in the PAR spectrum impaired seedling growth parameters under drought. When seedlings were cultivated under the intermediate spectrum (1.5BG1.5R), their development was significantly delayed: elongation of axial organs was restricted, and cotyledons remained poorly developed and unexpanded. These effects may be linked to shifts in metabolic pathways under water deficit, given that wheat plants grown under selective light spectra exhibit distinct physiological responses—blue light typically stimulates protein synthesis, whereas red light promotes carbohydrate synthesis [25,27].

The effect of melatonin on plant growth processes heavily depended on cultivation conditions. In darkness, melatonin exhibited a dual regulatory role in shoot morphogenesis: it shortened the hypocotyl (resembling a photomorphogenic response) while inhibiting cotyledon growth (resembling a skotomorphogenic response). Such responses suggest that melatonin influences both skoto- and photomorphogenesis by altering either endogenous melatonin levels or tissue sensitivity to the hormone. Melatonin enhanced the promoting effect of blue light on cotyledon growth (both length and width) and increased the dimensions of axial organs. Specifically, melatonin stimulated root and stem growth under 1.5BG1.5R and 2BGR, but inhibited root growth under BG2R (Figure 1a,b). In darkness, melatonin suppressed cotyledon blade growth, whereas under 2BGR it stimulated cotyledon expansion (Figure 1c). These findings indicate that the physiological outcome of melatonin treatment is strongly dependent on the proportion of blue light in the PAR spectrum. Overall, the observed effects demonstrate that the efficacy of exogenous melatonin in regulating morphogenesis is strictly conditioned by the lighting environment.

Root application of 1 µM melatonin alleviated the negative impacts of drought in darkness and under both the 2BGR and 1.5BG1.5R regimes. However, when the proportion of red light in the PAR spectrum was elevated (BG2R), melatonin exacerbated the inhibition of seedling growth. This phenomenon may be linked to phytochrome-dependent melatonin synthesis and signaling [28], as the highest endogenous melatonin levels have previously been reported in rice plants grown under combined red and blue light. Furthermore, drought tolerance under specific light spectra may be influenced by phytochrome-mediated pathways that control leaf development. For instance, a rice *phyB* mutant line exhibited reduced water loss and transpiration rates due to decreased leaf surface area, stomatal density, and stomatal dimensions [29].

Drought severely impairs the functioning of the photosynthetic apparatus through stomatal closure and reduced CO_2_ assimilation, alongside disruptions in chloroplast ultrastructure, chlorophyll biosynthesis, the uncoupling of electron transport from phosphorylation, and subsequent cellular ATP deficiency. Combined with the inhibition of the mitochondrial respiratory chain, these physiological bottlenecks ultimately suppress anabolic processes throughout the plant [30]. Therefore, we analyzed the morphology of the stomatal apparatus in cucumber plants grown under various light spectral regimes during drought exposure.

Short-term drought exposure (3 days) manifested as reductions in key growth parameters—including root and stem length, number of leaf tiers, and leaf surface area—alongside decreased stomatal density, smaller stomatal dimensions, stomatal closure (except under 2BGR), and elevated lipid peroxidation (LPO) intensity. The apparent increase in pigment concentrations under drought was likely driven by reductions in leaf surface area resulting from inhibited cell expansion and increased cellular compaction within the leaf [31,32]. Under the blue-enriched 2BGR regime, free proline accumulation was notably higher compared to treatments with a greater proportion of red light (1.5BG1.5R and BG2R). This aligns with findings in tomato plants, where blue light enhanced resistance to gray mold by stimulating free proline accumulation, polyphenolic content, and antioxidant enzyme activity [7]. Overall, shifts in light spectral composition modulated the drought-stress responses of cucumber plants by reprogramming underlying metabolic pathways. Interestingly, the least pronounced negative effects of drought were observed in plants cultivated under a higher proportion of red light (BG2R), whereas the most severe impacts occurred under the blue-enriched regime (2BGR).

Under red light, phytochromes are photoactivated and orchestrate numerous physiological processes across the plant life cycle, ranging from seed germination to flowering and fruiting. Furthermore, phytochromes regulate plant responses not only to light cues but also to various abiotic stressors, such as temperature extremes, salinity, and drought, all of which frequently trigger oxidative stress as a secondary effect. This cross-talk is primarily mediated by phytochrome regulation over the biosynthesis of low-molecular-weight antioxidants and photosynthetic pigments, as well as the transcriptional modulation of key cell-signaling genes [33].

The addition of 1 µM melatonin to the nutrient medium mitigated the adverse effects of drought by increasing root and stem length, leaf area, and the formation of new leaf tiers, while decreasing LPO intensity. Melatonin exhibited higher efficacy when plants were cultivated under BG2R and 2BGR.

Melatonin increased stomatal density per unit leaf surface in plants grown under BG2R, whereas it decreased this parameter under drought conditions in plants grown under 2BGR compared with both drought alone and the control (Table 2). The latter effect was most likely associated with the enhanced expansion of second-tier leaf surface area (Figure 4). Under drought, melatonin increased stomatal area and linear dimensions, thereby increasing stomatal aperture area in leaves formed under 2BGR and 1.5BG1.5R, but reduced these parameters under BG2R. The effects of melatonin on stomatal morphology under drought and 2BGR partially align with data obtained in rice plants, where melatonin treatment increased stomatal density and linear dimensions, including length, width, and total area [22]. The protective effect of melatonin under drought is likely associated with the maintenance of plant water relations, as melatonin has been demonstrated to preserve high turgor potential and relative water content in maize seedlings [34], thereby keeping stomata open under both optimal and water-deficit conditions. In our study, melatonin maintained stomata in a more open state under 2BGR, whereas under BG2R it promoted their partial closure, which was presumably aimed at limiting transpirational water loss.

Our findings demonstrate for the first time that light spectral composition is a decisive factor determining not only the efficacy but also qualitatively distinct strategies of melatonin-mediated protection in plants. These results designate melatonin as a light-dependent regulator of plant responses to drought, whose physiological targets depend heavily on photoreceptor activity and the spectral composition of PAR.

Under BG2R light, melatonin treatment altered the stomatal parameters of cucumber plants, which was accompanied by a decrease in the expression of *SNAT*, encoding serotonin N-acetyltransferase involved in melatonin biosynthesis. This downregulation of *SNAT* represents a distinct light-dependent molecular response and is consistent with published data showing the repression of *OsSNAT1* expression under red light—an effect mediated by phytochromes, given that the repressive action of red light was weakened or absent in phytochrome mutants [35,36].

The most pronounced protective effect of melatonin was observed under the light regime with an increased proportion of red light (BG2R). Under drought, melatonin induced a substantial recovery of photosynthetic parameters—specifically Y(II) and ETR—against the background of reduced LPO intensity and increased superoxide dismutase (SOD) activity. This corroborates data regarding the role of melatonin in protecting photosystem II and stabilizing the electron transport chain [21], particularly since red light can induce photooxidative stress during photosynthesis under drought conditions [37]. Furthermore, these findings align with observations in maize, where exogenous melatonin significantly enhanced photosynthetic activity and stimulated the antioxidant system (including SOD, CAT, and peroxidase activities), accompanied by reduced levels of H_2_O_2_ and malondialdehyde, as well as with studies in sorghum showing that melatonin preserves chloroplast ultrastructure and elevates antioxidant enzyme activity under water deficit [38,39].

Our RT-qPCR data indicate that, under a higher proportion of red light, melatonin induced the expression of antioxidant defense genes (*SOD* and *APX*) and the auxin response gene *ARF1* under drought conditions. Apparently, under red light, phytochromes modulate endogenous melatonin synthesis [28], whereas exogenous melatonin further reinforces this signal and shifts cellular metabolism toward enhanced enzymatic antioxidant defense. This response is particularly crucial under conditions of stomatal closure, where restricted CO_2_ assimilation increases the risk of photooxidative stress driven by excess light energy [37].

Notably, when plants were cultivated under red-enriched light (BG2R), melatonin treatment reduced *SNAT* expression. This result agrees with evidence that light of varying spectral quality modulates the expression of melatonin biosynthesis genes, including *SNAT* and *TDC* [28], as well as recent findings identifying *SNAT* as a direct transcriptional target of HY5, a central regulator of light signaling [40]. The reduction in *SNAT* expression under BG2R likely reflects negative feedback regulation in response to high melatonin accumulation, preventing its overproduction. Concurrently, the sharp induction of *ARF1* (encoding an auxin response factor) under the combined action of drought and melatonin under BG2R points to the active involvement of auxin signaling in mediating protection. As a structural analog of indole-3-acetic acid, melatonin can modulate auxin pathways [41], and transcriptomic analyses frequently confirm the melatonin-triggered activation of auxin-responsive genes [42].

Under the blue-enriched regime (2BGR), melatonin operated through a different strategy: it increased stomatal area and aperture width, maintaining stomatal openness, which is essential for gas exchange and transpiration. This corresponds with the well-established role of blue light as a master regulator of stomatal movements mediated by phototropin photoreceptors [43]. In contrast, under red-enriched light (BG2R), melatonin reduced stomatal aperture area, likely to restrict transpirational water loss. Additionally, an increase in SOD activity, MDA content, and *CAT* expression compared with the control suggests the induction of a melatonin-controlled oxidative burst. Such a redox signaling cascade may be associated with the activation of cryptochrome-dependent pathways [44], functioning as a preadaptive priming mechanism of the antioxidant system, a phenomenon documented for various chemical elicitors [45].

Conversely, under blue-enriched light (2BGR), melatonin alleviated drought stress primarily by optimizing stomatal apparatus geometry and enhancing guaiacol peroxidase activity. Although melatonin increased *CAT* expression under 2BGR drought conditions compared with the control, the combined stress and melatonin treatment actually reduced the transcriptional activity of *SOD*, *APX*, and *CAT* relative to drought treatment alone. This reduction may stem from melatonin functioning directly as an antioxidant, partially substituting for the enzymatic defense component and thereby lowering the metabolic and energetic costs associated with de novo enzyme synthesis [45].

Overall, this comprehensive study establishes that melatonin acts as a light-dependent regulator of stress tolerance, with the spectral composition of PAR serving as a switch between two distinct protective strategies. Light quality—specifically the ratio of red to blue spectral regions—is not merely an environmental background factor, but a critical determinant directing the efficacy and mechanisms of exogenous melatonin action under optimal and stress conditions. For the first time, we demonstrate that the protective role of melatonin under osmotic stress is executed through divergent strategies depending on the light regime, with maximum enhancement of drought tolerance in cucumber achieved under red-enriched light.

These findings strongly support the existence of a light-dependent framework for melatonin-mediated protection, which is consistent with evidence regarding photoreceptor involvement in the antioxidant effects of melatonin under light stress in *Arabidopsis thaliana* [42]. Thus, under a predominant red spectral region of PAR, a phytochrome-dependent pathway appears to be recruited, boosting antioxidant defenses and protecting the photosynthetic apparatus. In contrast, under blue-enriched light, the stomatal apparatus becomes the primary physiological target, ensuring the optimization of water status and gas exchange.

Several limitations of the present study should be acknowledged. First, PEG-induced osmotic stress does not fully replicate soil water deficit under natural or field conditions, as root hydraulic conductance, oxygen availability, nutrient mobility, and rhizosphere dynamics can differ substantially between hydroponic PEG solutions and natural soils. Second, our molecular analysis was restricted to selected target genes associated with antioxidant defense, melatonin biosynthesis, and auxin signaling, whereas transcripts directly related to ABA signaling, photoreceptor networks, ROS signaling, and broader drought-responsive transcriptional cascades were not evaluated. Third, the statistical evaluation relied on predefined pairwise comparisons within individual light regimes rather than testing main effects or factorial interactions among light quality, PEG treatment, and melatonin application. Consequently, the proposed signaling pathways and light-dependent response models should be viewed as working hypotheses that warrant further validation through comprehensive transcriptomic profiling, soil-based drought assays, and full factorial experimental designs.

## 4. Materials and Methods

### 4.1. Plant Material and Growth Conditions

Cucumber (*C. sativus* L.) plants of the early-maturing cultivar ‘Iziaschnyi’, obtained from the SeDeK agricultural company (Moscow, Russia), were used as the study material. The experiments were performed using both cucumber seedlings and actively growing vegetative plants.

Sterile cucumber seeds were sown on Murashige and Skoog (MS) medium (Papna industry Co., Ltd., Zhengzhou, China) [46] either without additives or supplemented with polyethylene glycol 6000 (PEG-6000) and/or melatonin, as described in Section 2.2. For each treatment and light regime, three independent culture vessels containing 20 seeds each were used. The vessels were transferred to the lighting units or kept in darkness for 7 days, after which root length, hypocotyl length, and the linear dimensions of the cotyledons were measured. Thus, each treatment included three biological replicates, with 20 seedlings per replicate.

For the experiment involving actively growing plants, cucumber seeds were germinated in trays containing vermiculite moistened with half-strength MS medium, placed in the lighting units, and cultivated for 9 days. The resulting seedlings were then transferred to individual containers on floating platforms containing half-strength MS medium and cultivated hydroponically for an additional 9 days. Hydroponic cultivation was selected to ensure uniform nutrient availability, precise control over the composition and osmotic potential of the growth medium, and uniform exposure of the root system to PEG-6000 and melatonin treatments.

Both seedlings and vegetative cucumber plants were grown under light with different combinations of three spectral regions of photosynthetically active radiation (PAR). Blue LEDs (SMD 3528) had an emission maximum at 440–460 nm, red LEDs (SMD 3528) had emission maxima at 630–660 nm, and white LEDs (SMD 3528) had a color temperature of 4000 K. The photon flux density ratios of blue:green:red light were 2:3:1 (designated as 2BGR), 1.5:3:1.5 (1.5BG1.5R), and 1:3:2 (BG2R). The incident photon flux density under all lighting conditions was maintained at 130–160 µmol m^−2^ s^−1^, with a 16 h photoperiod and a relative air humidity of 60–70%. The air temperature was kept at 25–27 °C.

### 4.2. Experimental Design and Treatments

Two experimental systems were used.

In the seedling experiment, sterile cucumber seeds were cultivated for 7 days on MS medium without additives (Control) or on medium supplemented with 8% PEG-6000 (PEG 8), 1 µM melatonin (1 µM Mel), or both 8% PEG-6000 and 1 µM melatonin (PEG 8 + 1 µM Mel). The seedlings were cultivated under the three PAR spectral regimes described above or in darkness.

In the experiment with actively growing plants, 18-day-old cucumber plants were divided into four treatment groups, with 20 plants per group, and cultivated for 3 days on media of different composition: (1) half-strength MS medium (Control); (2) half-strength MS medium supplemented with 8% PEG-6000 (PEG 8); (3) half-strength MS medium supplemented with 1 µM melatonin (1 µM Mel); and (4) half-strength MS medium supplemented with both 8% PEG-6000 and 1 µM melatonin (PEG 8 + 1 µM Mel). The experiment was independently repeated three times. At the end of the treatment period, the plants were 21 days old. The 3-day treatment period was selected to assess short-term physiological, biochemical, and transcriptional responses to PEG-induced osmotic stress in actively growing plants and to minimize the contribution of secondary effects associated with prolonged stress exposure.

The calculated osmotic potential of an 8% (*w*/*v*) PEG-6000 solution at 25–27 °C is approximately −0.11 to −0.12 MPa, according to the equation proposed by Michel and Kaufmann [47], corresponding to moderate osmotic stress conditions. The concentration of melatonin (1 µM) was selected based on previous studies demonstrating the physiological effectiveness of low micromolar concentrations of exogenous melatonin under abiotic stress conditions [15,16,17,18,19,20].

The selection of the PAR spectral regimes was based on published evidence showing light-dependent regulation of plant growth, photosynthetic processes, and melatonin metabolism. Phytochrome-mediated induction of melatonin synthesis was previously demonstrated in rice plants under cadmium stress, with the highest melatonin content observed under combined red and blue light [28]. Growth and organogenic processes in plants, including cucumber, are also known to depend on the spectral composition of radiation [31,48]. Furthermore, cucumber plants grown under red light without blue light exhibit disturbances in photosynthetic processes, whereas the addition of blue light restores the functioning of the photosynthetic apparatus [49].

### 4.3. Morphological and Stomatal Analysis

During the experiment, the linear dimensions of axial organs, cotyledon surface area, leaf surface area, and the structure of the abaxial epidermis were determined.

For 7-day-old seedlings, root length, hypocotyl length, cotyledon length, and cotyledon width were measured.

For 21-day-old vegetative plants, root length, stem length, the number of leaf tiers, and the surface area of first- and second-tier leaves were determined. The number of leaf tiers recorded immediately before the 3-day treatment was used as the initial baseline for evaluating subsequent shoot development.

The morphological characteristics of cucumber stomata were analyzed using a Micros microscope (Austria) on images captured with a digital camera. Measurements and parameter calculations were performed using Moticam 2300 software (Cabrera de Mar, Spain). The lower leaf surface was selected for analysis due to its lower pubescence. In cucumber plants of the cultivar ‘Iziaschnyi’, an anomocytic type of stomatal arrangement was observed.

Stomatal characteristics were determined using the epidermal imprint method described by Polachi-Molotkovsky [50]. From the resulting imprints, the following parameters were calculated: stomatal density per unit leaf surface area (1 mm^2^), stomatal aperture length and width, stomatal aperture area, stomatal length and width, total stomatal area, and the stomatal length-to-width ratio (L/W).

### 4.4. Determination of Photosynthetic Pigments

The content of photosynthetic pigments in ethanolic extracts was determined according to Lichtenthaler [51] and expressed as mg g^−1^ fresh weight. The optical density of the solutions was measured at 664 and 648 nm to determine chlorophyll *a* and chlorophyll *b*, respectively, and at 470 nm to determine carotenoid content. Solution turbidity was measured at 720 nm.

### 4.5. Determination of Total Phenolic Compounds

The total content of phenolic compounds was determined using the Folin-Denis reagent according to the method of Swain and Hillis [52].

### 4.6. Chlorophyll Fluorescence Measurements

The photochemical activity of the photosynthetic apparatus was measured using a MINI-PAM-II fluorometer (Heinz Walz, Effeltrich, Germany). Fluorescence parameters were recorded after 20 min of dark adaptation of intact cucumber leaves.

The following parameters were determined: maximum photochemical quantum yield of photosystem II (Fv/Fm), effective quantum yield of PSII photochemistry (Y(II)), non-photochemical quenching (NPQ), quantum yield of regulated non-photochemical energy dissipation (Y(NPQ)), quantum yield of non-regulated non-photochemical energy dissipation (Y(NO)), relative electron transport rate (ETR), photochemical quenching coefficients (qP and qL), and the non-photochemical quenching coefficient qN [53,54]. Fluorescence quenching coefficients and the relative electron transport rate were calculated using MINI-PAM-II software (current version 3.40).

### 4.7. Determination of Osmotic Adjustment and Oxidative Stress Markers

Lipid peroxidation (LPO) intensity was assessed spectrophotometrically by measuring malondialdehyde (MDA), a product of lipid peroxidation. MDA content was determined from the absorbance of the colored complex formed during its reaction with thiobarbituric acid upon heating [55].

To evaluate osmotic adjustment and non-enzymatic antioxidant defence, free proline content was determined. Free proline was extracted and quantified according to Bates et al. [56], based on the formation of a red-colored product in the reaction of an extract aliquot with ninhydrin under acidic conditions.

### 4.8. Antioxidant Enzyme Activity

The activities of antioxidant enzymes, namely superoxide dismutase (SOD, EC 1.15.1.1) and guaiacol peroxidase (GPOX, EC 1.11.1.7), were determined according to previously described methods [12].

Total SOD activity was measured using a reaction mixture containing 100 μL of supernatant, 1.75 mL of 50 mM Tris–HCl buffer (pH 7.8), 0.2 mL of 0.1 M DL-methionine, 0.063 mL of 1.7 mM nitroblue tetrazolium, 0.047 mL of 1% Triton X-100, and 0.060 mL of 0.004% riboflavin (Fermentas, Nacogdoches, TX, USA). The reaction was carried out under LED illumination at a photon flux density of 232 μmol m^−2^ s^−1^ for 30 min. Absorbance was measured at 560 nm using a Genesys 10S UV–Vis spectrophotometer (Thermo Scientific, Waltham, MA, USA).

To assess GPOX activity, the reaction mixture contained 50 μL of supernatant, 1.95 mL of 0.066 M potassium phosphate buffer (pH 7.4), 200 μL of 7 mM guaiacol, and 200 μL of 0.01 M H_2_O_2_. Absorbance was measured at 470 nm using a Genesys 10S UV–Vis spectrophotometer (Thermo Scientific, MA, USA).

Protein concentration in the enzyme extracts was determined according to Esen [57].

### 4.9. RNA Isolation, Target Gene Selection, and RT-qPCR Analysis

Leaf tissues were collected from 21-day-old cucumber plants after the 3-day treatment period. The tissues were ground in liquid nitrogen, and total RNA was extracted using TRIzol reagent (Invitrogen, Waltham, MA, USA) according to the manufacturer’s instructions. Genomic DNA contamination was removed by treatment with RNase-free DNase I (Thermo Fisher Scientific, Waltham, MA, USA).

cDNA synthesis was performed using MMLV RevertAid reverse transcriptase (Thermo Fisher Scientific, MA, USA) and an oligo(dT)_21_ primer (Evrogen, Moscow, Russia).

Target genes were selected according to their functional involvement in the physiological processes examined in this study. The selected genes represented antioxidant defence (*SOD*, *POD*, *APX*, and *CAT*), melatonin biosynthesis (*ASMT*, *TDC1*, *T5H*, and *SNAT*), and auxin-mediated regulation (*ARF1*).

Primer sequences for the target genes were selected based on cucumber nucleotide sequences available in the NCBI database using Primer-BLAST 20 and Vector NTI 11.0. The databases and software were accessed on 20 February 2025. Primer sequences are presented in Table S1. The cucumber β-actin gene (*Act-7*), previously validated as a stable reference gene for this species, was used to normalize target gene expression levels [21,58].

The corresponding amplicon sizes are also presented in Table S1.

RT-qPCR analysis was performed using a CFX96 Touch Real-Time PCR Detection System (Bio-Rad, Hercules, CA, USA). The reaction mixture was prepared using qPCRmix HS SYBR (Eurogen, Moscow, Russia), gene-specific primers, ddH_2_O, and cDNA samples according to the manufacturer’s instructions.

Each reaction was performed in three technical replicates. Three independent biological replicates were analyzed. The cucumber β-actin reference gene was used to normalize target gene expression levels.

Standard curves were generated using serial 10-fold dilutions of cDNA samples. Amplification specificity was verified by melting-curve analysis, which showed a single peak for each primer pair. Primer amplification efficiency was calculated from the slopes of the standard curves. Relative gene expression was calculated using the 2^−ΔΔCt^ method.

### 4.10. Statistical Analysis

The seedling experiment included three independent culture vessels per treatment and light regime, with 20 seedlings in each vessel. The vegetative-plant experiment included 20 plants per treatment in each of three independent experimental runs. For biochemical analyses, 5–9 biological samples were analyzed per treatment, depending on the parameter. RT-qPCR measurements were performed using three independent biological replicates, each analyzed in three technical replicates.

Statistical significance was assessed using a two-sided Student’s *t*-test for a restricted set of biologically defined pairwise comparisons. Within each light regime, each treatment was compared with the corresponding control, and PEG 8 + 1 μM Mel was compared with PEG 8 to evaluate the effect of melatonin under PEG-induced osmotic stress. Exhaustive comparisons among all possible treatment combinations were not performed. This approach was selected because the study focused on these specific within-regime contrasts rather than on the formal estimation of the main effects and higher-order interactions among light regime, PEG treatment, and melatonin application. Accordingly, the observed light-dependent response patterns were interpreted cautiously and were not considered formal statistical interactions. Differences were considered statistically significant at *p* ≤ 0.05.

## 5. Conclusions

For the first time, this study establishes that the spectral composition of photosynthetically active radiation (PAR) is not merely a background environmental factor, but a critical modulator determining the strategies and physiological targets of exogenous melatonin-mediated protection in cucumber plants under osmotic stress. Specifically, under a red-enriched light regime (BG2R), melatonin (1 µM) appears to engage phytochrome-dependent pathways, thereby enhancing antioxidant responses and protecting the photosynthetic apparatus. Under these conditions, melatonin restores PSII photochemical activity [Y(II) and ETR], reduces lipid peroxidation intensity, upregulates the expression of antioxidant defense genes (*SOD* and *APX*) and the auxin response gene *ARF1*, and downregulates the melatonin biosynthesis gene *SNAT*. Conversely, under blue-enriched light, the stomatal apparatus serves as the primary target: melatonin maintains stomatal openness, increases stomatal area and density, promotes proline accumulation, and enhances peroxidase activity, thereby minimizing water loss and sustaining gas exchange. Collectively, these results demonstrate that melatonin acts as a light-dependent regulator of drought stress responses in cucumber, with its physiological and molecular outcomes strictly governed by the PAR spectrum through interactions with core photoreceptor systems, including phytochromes, cryptochromes, and phototropins. These findings open new avenues for elucidating the integrative mechanisms underlying the cross-talk between light signaling and hormonal regulation in plants, offering promising tools for developing advanced agrotechnological strategies to enhance crop drought tolerance under controlled environment agriculture.

## Figures and Tables

**Table 1:** Effect of melatonin on stomatal density and morphology in second-tier leaves of 21-day-old *C. sativus* plants exposed to PEG-induced drought under different light spectral compositions.

Parameter	Light Regime	Control	1 μM Mel	PEG 8	PEG 8 + 1 μM Mel
Stomatal density (stomata mm^−2^)	2BGR	214.5 ± 5.8	217.1 ± 6.4	188.0 ± 7.6 *	166.7 ± 5.0 *^
	1.5BG1.5R	262.4 ± 14.9 #	226.1 ± 6.4	196.6 ± 7.3 *	199.4 ± 9.5 *
	BG2R	153.8 ± 4.9 #	262.8 ± 12.8 *	131.1 ± 5.1 *	274.5 ± 11.3 *^
Stomatal aperture length (μm)	2BGR	23.99 ± 0.60	22.06 ± 1.01	21.54 ± 0.60	24.42 ± 0.46
	1.5BG1.5R	18.1 ± 0.3	24.3 ± 0.4 *	19.1 ± 0.7	23.1 ± 0.9
	BG2R	23.5 ± 0.7	20.4 ± 1.1	25.2 ± 0.7	20.8 ± 0.5
Stomatal aperture width (μm)	2BGR	8.29 ± 0.22	8.39 ± 0.33	4.91 ± 0.29 *	10.15 ± 0.28
	1.5BG1.5R	6.0 ± 0.3	9.9 ± 0.3 *	5.3 ± 0.4	8.1 ± 0.4
	BG2R	7.8 ± 0.3	7.6 ± 0.4	8.5 ± 0.4	7.2 ± 0.3
Stomatal aperture area (μm^2^)	2BGR	157.22 ± 6.04	148.46 ± 11.6	84.20 ± 5.96 *	195.17 ± 6.73 *^
	1.5BG1.5R	86.7 ± 4.9	191.8 ± 7.5 *	80.5 ± 7.8	148.6 ± 12.0 *^
	BG2R	145.3 ± 9.1	123.4 ± 11.2	171.4 ± 10.5 *	119.1 ± 5.5 *^
Stomatal area (μm^2^)	2BGR	410.1 ± 14.3 #	378.5 ± 29.0	327.6 ± 21.0 *	590.9 ± 23.2 *^
	1.5BG1.5R	237.5 ± 8.8 #	421.8 ± 11.6 *	281.1 ± 17.1 *	417.8 ± 30.6 *^
	BG2R	344.2 ± 21.4 #	295.6 ± 29.6	525.1 ± 26.3 *	358.5 ± 15.1 ^

Note: 2BGR, blue:green:red light = 2:3:1; 1.5BG1.5R, blue:green:red light = 1.5:3:1.5; BG2R, blue:green:red light = 1:3:2. * indicates statistically significant differences compared with the corresponding control within the same light regime (*p* ≤ 0.05); ^ indicates statistically significant differences compared with PEG 8 within the same light regime (*p* ≤ 0.05); # indicates statistically significant differences among light regimes within the control treatment (*p* ≤ 0.05).

**Table 2:** Effect of melatonin on PSII photochemical activity in leaves of 21-day-old *C. sativus* plants under drought stress (PEG 8) and light of different spectral compositions.

Light Type	Treatment	Y(II)	ETR	qP	qN	qL	NPQ
2BGR	Control	0.43 ± 0.02	34.40 ± 1.20	0.56 ± 0.02	0.43 ± 0.01	0.23 ± 0.01	0.59 ± 0.01
	1 μM Mel	0.44 ± 0.02	34.70 ± 1.40	0.58 ± 0.03	0.45 ± 0.03	0.26 ± 0.03	0.66 ± 0.07
	PEG 8	0.38 ± 0.03	30.20 ± 2.30	0.50 ± 0.05	0.42 ± 0.08	0.20 ± 0.05	0.60 ± 0.18
	PEG 8 + 1 μM Mel	0.43 ± 0.02	34.60 ± 1.48	0.59 ± 0.04	0.47 ± 0.04	0.27 ± 0.04	0.70 ± 0.09
1.5BG1.5R	Control	0.41 ± 0.01	33.05 ± 1.15	0.54 ± 0.02	0.40 ± 0.05	0.22 ± 0.02	0.53 ± 0.11
	1 μM Mel	0.44 ± 0.02	35.17 ± 1.51	0.60 ± 0.04	0.43 ± 0.05	0.28 ± 0.04	0.61 ± 0.11
	PEG 8	0.38 ± 0.03	29.90 ± 2.00	0.50 ± 0.04	0.41 ± 0.08	0.2 ±0.03	0.56 ± 0.18
	PEG 8 + 1 μM Mel	0.40 ± 0.01	32.10 ± 0.70	0.54 ± 0.01	0.43 ± 0.02	0.22 ± 0.01	0.59 ± 0.05
BG2R	Control	0.38 ± 0.01	30.53 ± 0.83	0.51 ± 0.02	0.42 ± 0.02	0.20 ± 0.01	0.57 ± 0.05
	1 μM Mel	0.40 ± 0.01	32.10 ± 0.96	0.56 ± 0.02	0.48 ± 0.03	0.26 ± 0.02	0.71 ± 0.07 *
	PEG 8	0.37 ± 0.02	29.55 ± 1.45	0.50 ± 0.03	0.40 ± 0.07	0.20 ± 0.03	0.55 ± 0.15
	PEG 8 + 1 μM Mel	0.50 ± 0.01 *^	39.87 ± 0.88 *^	0.67 ± 0.02 *^	0.44 ± 0.01	0.34 ± 0.02	0.62 ± 0.01

Note: * indicates statistically significant differences compared with the corresponding control within the same light regime (*p* ≤ 0.05); ^ indicates statistically significant differences compared with PEG 8 within the same light regime (*p* ≤ 0.05).

## Data Availability

The data presented in this study are available on request from the corresponding author.

## Supplementary Materials

The following supporting information can be downloaded at: https://www.mdpi.com/article/10.3390/ijms27156898/s1.

## Abbreviations

ROS, reactive oxygen species; GPOX, guaiacol peroxidase (EC 1.11.1.7); cDNA, complementary DNA; MDA, malondialdehyde; MS medium, Murashige and Skoog medium; PEG, polyethylene glycol; LPO, lipid peroxidation; SOD, superoxide dismutase (EC 1.15.1.1); PAR, photosynthetically active radiation; 2BGR, lighting with a blue:green:red PAR ratio of 2:3:1; 1.5BG1.5R, lighting with a blue:green:red PAR ratio of 1.5:3:1.5; BG2R, lighting with a blue:green:red PAR ratio of 1:3:2; APX, ascorbate peroxidase; *ARF*, auxin response factor; *ASMT*, acetylserotonin O-methyltransferase; CAT, catalase; ETR, relative electron transport rate; Fv/Fm, maximum photochemical quantum yield of PSII; NPQ, non-photochemical fluorescence quenching; POD, peroxidase; qL and qP, photochemical quenching coefficients; RT-qPCR, reverse transcription quantitative PCR; *SNAT*, serotonin N-acetyltransferase; TDC, tryptophan decarboxylase; T5H, tryptamine 5-hydroxylase; Y(II), effective quantum yield of PSII photochemistry; Y(NO), quantum yield of non-regulated non-photochemical energy dissipation; Y(NPQ), quantum yield of regulated non-photochemical energy dissipation.
