# Supplementary material for: Light-Dependent Regulation of Drought Tolerance in Cucumber Plants by Melatonin
Source: Int J Mol Sci. 2026 Aug 1;27(15):6898. doi: 10.3390/ijms27156898 (PMC13467344; doi:10.3390/ijms27156898)
Supplement: Supplementary file 1 [file ijms-27-06898-s001.zip › ijms-4444800-supplementary.pdf]

Table S1. Primers used for RT-qPCR.

№	Gene Name	Function	5′→3′ Forward Primer Sequence	5′→3′ Reverse Primer Sequence	Amplicon Size (bp)
		Constitutive reference genes			
1	*Act-7* (Actin)	Cucumber *β*-actin gene	CCA CGA AAC TAC TTA CAA CTC CAT C	GGG CTG TGA TTT CCT TGC TC	137
		Genes encoding antioxidant defense enzymes			
2	*SOD* (Superoxide Dismutase)	Cu/Zn-superoxide dismutase-like protein, transcript variant X2	GAA AGA TGG TGA AGG CTG TGG	GCA CCA TGT TGT TTT CCA GCA G	220
3	*POD* (Peroxidase)	Peroxidase	GGT TTC TAT GCC AAA AGC TGC CC	CAG CTT GGT TGT TTG AGG TGG AG	178
4	*APX* (Ascorbate Peroxidase)	Cytosolic-like L-ascorbate peroxidase	GTG CTA CCC TGT TGT GAG TG	AAC AGC GAT GTC AAG GCC AT	226
5	*CAT* (Catalase)	Catalase isozyme 3	AAT GGC CGG AGG ATG TGA	CCA ACG ACA TAG AGA AAG CCA AC	111
		Genes associated with plant hormone biosynthesis pathways and transcription factors			
6	*ASMT* (Acetylserotonin Methyltransferase)	Acetylserotonin O-methyltransferase	ATT GGA AGT TTA GTT GAT GTG GGA	AGC ATC AGC CTT GGG AAT GGA AT	183
7	*TDC1* (Tryptophan Decarboxylase)	Tyrosine/DOPA decarboxylase 1	ATA AAT GGT TCT TCT CGG CGC CAG	ACC CGA ATC AGT TGC CCT A	119
8	*T5H* (Tryptamine 5-Hydroxylase)	Tryptamine 5-hydroxylase	AGC TTG TGC AGG CTA CCA ACT	GAA CGT TGG AAC AAA CTT GTG	182
9	*SNAT* (Serotonin N-Acetyltransferase)	Acetyltransferase	AGT CCC CTG TTT CAG AGG AGA AT	AGA TTC CGA TAA AAC TCT ACC AC	232
10	*ARF1* (Auxin Response Factor)	Auxin response factor	CCA GAT CCT CCC CTT CCT GA	GTC ATC CGC ATG CCT CCT AA	120
